# Isolated Lateral Malleolar Fracture Treated with a Bioabsorbable Magnesium Compression Screw

**DOI:** 10.7759/cureus.2539

**Published:** 2018-04-26

**Authors:** Baver Acar, Melih Unal, Adil Turan, Ozkan Kose

**Affiliations:** 1 Department of Orthopaedics and Traumatology, University of Health Sciences, Medical Faculty, Antalya Education and Research Hospital, Antalya, Turkey

**Keywords:** magnesium, ankle fracture, bioabsorbable screw, lateral malleolar fracture

## Abstract

Magnesium (Mg) bioabsorbable screws are new biomaterials used in fracture fixation. In the current literature, there is only one case report on the use of magnesium bio-absorbable screws in ankle fractures. Within the present study, a 19-year-old female who sustained an isolated lateral malleolar fracture was treated with open reduction and intramedullary Mg screw fixation and then followed up for two years. Fracture union was achieved without any complication such as failure of fixation, loss of reduction, infection, or any other adverse reaction. Mg bioabsorbable screws are an alternative method of fracture fixation as compared to conventional metallic implants since they eliminate the need for implant removal.

## Introduction

Isolated lateral malleolar fractures, which are the most common types of fractures seen around the ankle joint, constitute 70% of all ankle fractures [1]. These fractures are classified using the AO-Weber system according to the location of the fracture and the integrity of the syndesmosis. Basically, there are three types of lateral malleolar fractures, known as the Weber type A (infrasyndesmotic), Weber type B (trans-syndesmotic), and Weber type C (suprasyndesmotic). Weber type A fractures are considered stable fractures since the ankle syndemosis remains intact and non-displaced fractures can be treated conservatively with cast immobilization. However, surgical fixation is indicated in displaced fractures that cause articular incongruity (step-off) and shortening of the fibula [2].

Currently, various surgical fixation techniques are used for infrasyndesmotic lateral malleolar fractures, but these fractures are best fixed using either tension band wire or a small oblique intramedullary screw. Since the soft tissue and skin envelope around the lateral malleolus is poor, early complications such as wound healing and infection can be seen. Furthermore, in the later period, the metallic implants may necessitate a removal operation in cases of pain and skin irritation and difficulty in wearing shoes [3]. In a study that investigated the necessity for implant removal after ankle fractures in a large series of patients (n=997), the rate of implant removal was reported to be as high as 17% [4].

Therefore, the use of bioabsorbable implants may provide an advantage in eliminating the need for secondary implant removal interventions in cases of lateral malleolus fractures. Previously, plates and screws made from polymers such as polyglycolic acid or polylactic acid have been reported for the fixation of ankle fractures, with successful results. However, sterile sinus formation, foreign body reactions, and granuloma formation related to these implants have been reported [5]. Technological developments in bioabsorbable materials are ongoing and new biomaterials are currently being developed. One of these materials is magnesium along with its alloys. In fact, the idea of using biomaterials manufactured from magnesium is not new, as it was first used in the field of orthopedics in the 1900s for ostheosynthesis. During its initial application, the rapid corrosion of magnesium caused an unexpected clinical picture and, therefore, use was suspended for a long period of time [6].

However, the manufacture of new magnesium alloys, such as MgYREZr, with improved corrosion resistance, has opened new horizons for its use in orthopedics and fracture fixation. Cannulated headless compression screws made of magnesium (alloy MgYREZr) have been available on the market for ostheosynthesis since 2013 [3]. To date, there have been a few clinical trials although several experimental animal trials have been performed on this innovative biomaterial. To the best of our knowledge, there has been only one case report regarding the use of the MgYREZr-based screws in ankle fractures. In this paper, a further case is presented of a patient with an isolated lateral malleolus fracture treated with a magnesium compression screw and our clinical experience is reported.

## Case presentation

**Figure 1:**
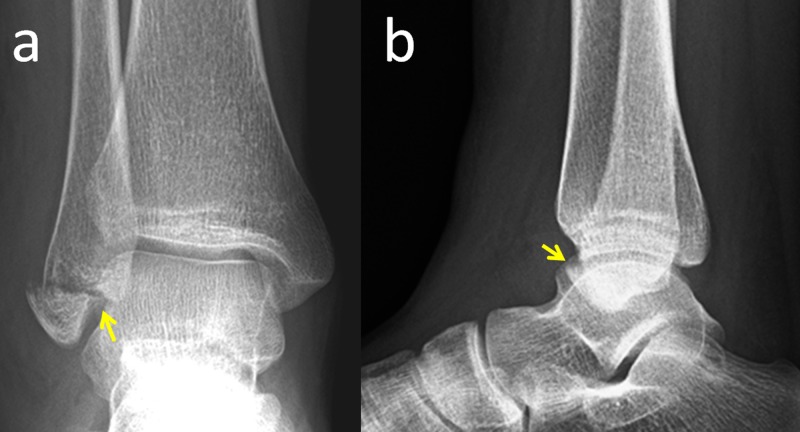
Preoperative radiographs Preoperative anteroposterior (a) and lateral (b) ankle radiographs of the patient. Yellow arrows show the distal fibular fracture.

A 19-year-old female was admitted to the emergency department with complaints of pain and swelling on the lateral side of the ankle after sustaining an ankle sprain. The patient was unable to bear weight upon admission. On physical examination, there was prompt swelling over the lateral side of the ankle and the tip of the fibula was tender on palpation. The ankle's range of motion was limited. The neurovascular examination was normal and the direct radiographic examination revealed a displaced distal fibular fracture (Weber type A) (Figure 1). As the fracture was intra-articular and there was considerable displacement (>4 mm), fixation of the fracture was mandatory.

**Figure 2:**
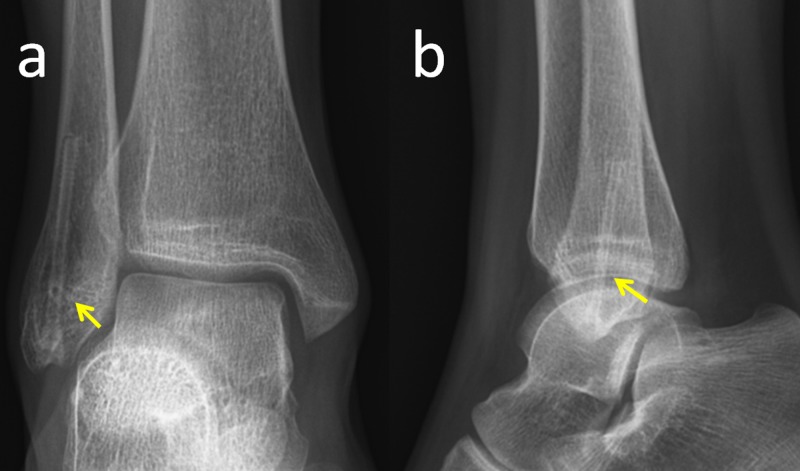
Postoperative radiographs Final ankle radiographs of the patient, two years after the operation. Yellow arrows show the shadow of the screw.

Under spinal anesthesia and tourniquet control, a small longitudinal incision was made over the distal fibula. The fracture was reduced and fixed with a single, 3.2 mm, intramedullary, magnesium headless compression screw (MAGNEZIX CS, Syntellix AG, Hannover, Germany) in a retrograde manner from the tip of the fibula. A short-leg plaster cast was applied to the patient for four weeks. After the removal of the cast, full weight-bearing was encouraged and ankle joint exercises were started. During the follow-up, fracture union was achieved without any complications within eight weeks (Figure 2).

**Figure 3:**
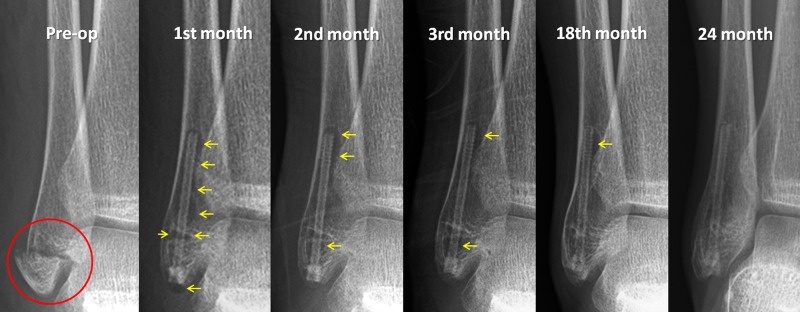
Serial radiographic examination Serial radiographic follow-up of the patient. Note the gradual reduction of the radiolucent zone around the screw at each subsequent follow-up examination up to 24 months. (The red circle shows the fracture and the yellow arrows show the radiolucent zones around the screw)

At the final follow-up examination, two years after the operation, the American Orthopaedic Foot & Ankle Society (AOFAS) score was 100 points and the patient had returned to the pre-injury level of activity. During the serial radiographic follow-up, a radiolucent zone was seen around the screw, but on the final follow-up radiograph, this radiolucency had almost completely disappeared (Figure 3).

## Discussion

The ideal fracture fixation material is expected to provide sufficient primary strength to keep the fracture fragments safe and should preserve this stability during the course of fracture union, without interfering with the normal fracture healing processes [7]. In the current case, the fracture union was achieved uneventfully without displacement throughout the follow-up period. Although it is difficult to statistically claim that this implant provides adequate fixation based on a single case experience, magnesium screws may be an alternative fixation method in fracture treatment.

The use of magnesium bioabsorbable screws in orthopedic surgery began with the fixation of distal metatarsal osteotomies for hallux valgus deformity correction. In these initial studies, no significant difference was found between magnesium versus titanium screws [8-10]. However, there are limited clinical data about the use of Mg bioabsorbable screws in trauma surgery, particularly on larger bones or joints. Biber et al. described a patient with an osteochondral fracture of the humeral capitellum treated with a magnesium screw, which resulted in fracture union without any complications [11]. Another trauma case with a lateral malleolar avulsion fracture was treated with a magnesium screw and fracture union was obtained within three months [3]. With the use of magnesium-based screws in five patients with six displaced mandibular condylar head fractures, Leonhardt et al. reported satisfactory functional results without a further need to remove the implants [12]. Similar to these previous findings and reports, fracture union was obtained in the current case without any clinical problems. However, some radiological findings were observed that may not be familiar to all orthopedic surgeons.

A radiolucent zone surrounding the implants was observed on the radiographs in this patient. This zone appeared to be most prominent in the first postoperative month and was seen to gradually reduce at each of subsequent follow-up examinations. By the 18th month postoperatively, it had almost completely disappeared. At the final follow-up, 24 months after the surgery, the magnesium screw could still be detected but only as a weak silhouette. Although a radiolucent zone around the screw suggests that the screw may be loose, no displacement or loss of reduction occurred despite early weight bearing in the fourth week, and the initiation of physical exercises. This radiographic radiolucency has also been reported in previous studies. Meier and Panzica reported the results of five patients with scaphoid fracture treated with magnesium screw fixation. Taking computed tomography (CT) scans and x-rays as a basis, they speculated about “cyst formation” in the early postoperative period and claimed delayed union in three out of the five patients. However, on the follow-up radiographs, union was seen to have been achieved in all patients and functional scores were excellent in all cases. Despite the final fracture union and excellent clinical functions, the authors did not recommend the use of magnesium screws on the basis of this radiological phenomenon [13]. Radiolucencies can be explained by the degradation process; in other words, corrosion, of the implant. From our experience, regular bone healing was not affected in the current case. Surgeons who use this innovative biomaterial should be aware of these radiological findings and should focus more on fracture union, loss of reduction, and failure of the fixation. Furthermore, the correlation between clinical findings and radiological images should be carefully evaluated.

## Conclusions

In this case of a young adult with an isolated lateral malleolar fracture, a successful treatment outcome was obtained with magnesium screws as a new bioabsorbable implant. Despite the radiolucency around the screw, and even the early weight bearing of the patient at four weeks, no failure of the implant or re-displacement of the fracture was observed, and there was no interference with the early phases of fracture union or final consolidation. Magnesium screws seem to be appropriate candidates for an alternative fracture fixation method. However, further clinical trials on larger numbers of patients are preferable for this special indication.
